# Catalytic Conversion of *n*-C_7_ Asphaltenes and Resins II into Hydrogen Using CeO_2_-Based Nanocatalysts

**DOI:** 10.3390/nano11051301

**Published:** 2021-05-14

**Authors:** Oscar E. Medina, Jaime Gallego, Sócrates Acevedo, Masoud Riazi, Raúl Ocampo-Pérez, Farid B. Cortés, Camilo A. Franco

**Affiliations:** 1 Grupo de Investigación en Fenómenos de Superficie—Michael Polanyi, Departamento de Procesos y Energía, Facultad de Minas, Universidad Nacional de Colombia, Sede Medellín, Medellín 050034, Colombia; oemedinae@unal.edu.co; 2 Química de Recursos Energéticos y Medio Ambiente, Instituto de Química, Universidad de Antioquia UdeA, Medellín 050010, Colombia; andres.gallego@udea.edu.co; 3 Facultad de Ciencias, Escuela de Química, Universidad Central de Venezuela, Caracas 1040, Venezuela; socrates.acevedo@gmail.com; 4 Enhanced Oil Recovery Research Center, IOR-EOR Research Institute, Shiraz University, Shiraz 7193616511, Iran; mriazi@shirazu.ac.ir; 5 Centro de Investigación y Estudios de Posgrado, Facultad de Ciencias Químicas, Universidad Autónoma de San Luis Potosí, San Luis Potosí 78210, Mexico; raul.ocampo@uaslp.mx

**Keywords:** adsorption, asphaltene-resins mixtures, hydrogen production, nanocatalysts, steam catalytic gasification

## Abstract

This study focuses on evaluating the volumetric hydrogen content in the gaseous mixture released from the steam catalytic gasification of *n*-C_7_ asphaltenes and resins II at low temperatures (<230 °C). For this purpose, four nanocatalysts were selected: CeO_2_, CeO_2_ functionalized with Ni-Pd, Fe-Pd, and Co-Pd. The catalytic capacity was measured by non-isothermal (from 100 to 600 °C) and isothermal (220 °C) thermogravimetric analyses. The samples show the main decomposition peak between 200 and 230 °C for bi-elemental nanocatalysts and 300 °C for the CeO_2_ support, leading to reductions up to 50% in comparison with the samples in the absence of nanoparticles. At 220 °C, the conversion of both fractions increases in the order CeO_2_ < Fe-Pd < Co-Pd < Ni-Pd. Hydrogen release was quantified for the isothermal tests. The hydrogen production agrees with each material’s catalytic activity for decomposing both fractions at the evaluated conditions. CeNi1Pd1 showed the highest performance among the other three samples and led to the highest hydrogen production in the effluent gas with values of ~44 vol%. When the samples were heated at higher temperatures (i.e., 230 °C), H_2_ production increased up to 55 vol% during catalyzed *n*-C_7_ asphaltene and resin conversion, indicating an increase of up to 70% in comparison with the non-catalyzed systems at the same temperature conditions.

## 1. Introduction

Nowadays, global warming and energy supply challenges have attracted increasing attention worldwide [1]. Hydrogen is a renewable and clean energy, and its application only generates water as by-product. However, fossil fuels are currently the most used sources for energy consumption [2]. Generally, there is a desire to generate an energy transition from fossil fuels to a cleaner energy source, which include the use of hydrogen [3]. During the energy transition, co-production of fossil fuels and hydrogen could be an exciting strategy to supply several issues.

As hydrocarbons are hydrogen-rich compounds, they can be used to produce hydrogen by applying different methods such as gasification with air and oxygen, and steam reforming [4]. The first two methods have been extensively studied in coal gasification processes and natural gas reforming [5,6]; however, hydrogen production from crude oils emerges as a novel research topic with many challenges and opportunities. Crude oils are classified into light (LO), medium (MO), heavy (HO), and extra heavy (EHO) oils [7]. HO and EHO represent about 70% of the worldwide oil reserves. They are characterized by high viscosities and low American Petroleum Institute (API) gravity values, mainly associated with the high content of asphaltenes and resins [8,9,10,11,12]. Asphaltenes and resins are complex molecules composed of polyaromatic hydrocarbons cores surrounded by aliphatic chains [13,14,15,16]. Resins have a lower amount of aromatic species but longer aliphatic chains; therefore, they have a higher H/C ratio and lower polarity than asphaltenes [17,18]. Commonly, to improve the mobility of HO and EHO, thermal processes like steam injection [19,20], steam-assisted gravity drainage (SAGD) [7,21], and in situ combustion [22] are used. However, in most cases, these techniques produce less than 50% in situ crude oil and low calorific gaseous products, including greenhouse gases like CO_2_ [23,24,25,26,27,28].

Other nanoparticles and nanofluid-based technologies have been used to increase the efficiency of the thermal process. Some studies report that nanocatalysts have great potential to improve thermal methods efficiency by decomposing asphaltenes and upgrading crude oil [29,30,31,32]. CeO_2_ has been widely used for assisting several reactions and processes [33,34] as this catalyst has a fluorite cubic structure and its cations migrate easily at low temperatures [35]. The cerium ion circulates between Ce^4+^ and Ce^3+^ through a redox cycle; therefore, the material surface can store oxygen [36]. To improve the nanocatalysts adsorptive and catalytic performance, some authors proposed the use of functionalized and/or composite nanomaterials [29,37,38,39,40]. Therefore, nanocrystals of transition (NOT) and noble element oxides (NON) supported over different nanocatalysts, including CeO_2_ have been used, obtaining satisfactory results.

Previously, we developed a study to evaluate the effect of three bi-elemental systems, which composed of 1.0% in mass fraction of Ni-Pd, Co-Pd, and Fe-Pd supported on for the steam catalytic gasification of *n*-C_7_ asphaltenes [38]. The systems were named as CeNi1Pd1, CeCo1Pd1, and CeFe1Pd1, respectively. The results demonstrate higher *n*-C_7_ asphaltene adsorption for CeNi1Pd1 nanocatalysts, followed by CeCo1Pd1, CeFe1Pd1, and CeO_2_ [38]. During the thermal experiments, it was observed that two of the primary gaseous products (i.e., CO and CH_4_) were released. CO can be used to develop water-gas shift (WGS) reaction (Equation (1)) as well as CH_4_ for steam reforming (Equation (2)). It is worth mentioning that steam as a gasifying agent is necessary to convert the gas products into hydrogen-enriched synthesis gas and applied WGS. However, the release of hydrogen was not analysed in this work.

(1) $$\mathrm{CO}\;+\;H_{2}O⇌\;{\mathrm{CO}}_{2}+\;H_{2}$$

(2) $${\mathrm{CH}}_{4}+\;H_{2}O⇌\mathrm{CO}\;+\;{3H}_{2}$$

Due to the potential application of this type of nanocatalysts, it is expected that they can be used in the reservoir for co-production of upgraded crude oils and hydrogen through steam catalytic gasification [2]. To date, there are no works reported in the literature evaluating this technology. Since crude oil is a complex mixture of different fractions, it is important to evaluate the hydrogen production from the steam catalytic gasification of *n*-C_7_ asphaltenes, resins II, and combinations of them. Therefore, this work aims to evaluate for the first time the hydrogen production efficiency of different CeO_2_ based- nanocatalysts containing different ratios between three NOT (i.e., Ni, Co, and Fe) and a NON element oxide (Pd) through the steam catalytic gasification of the crude oil heaviest fractions (i.e., resins II and *n*-C_7_ asphaltenes). The adsorption of both fractions and a mixture of them in the nanocatalysts is evaluated to meet this objective. Then, the hydrogen releasing was calculated during isothermal gasification process. Some variables such as temperature, resins:asphaltenes (R:A) ratio, and nanocatalyst nature were analyzed. This study is expected to broaden the use of fossil fuels for producing a hydrogen-rich gas with low content of CO_2_ (<5% vol) from the gasification of the most complex fractions of crude oil.

## 2. Materials and Methods

### 2.1. Materials 

CeNi1Pd1, CeFe1Pd1, and CeCo1Pd1 were previously synthesized through the incipient wetness technique [41] using commercial cerium oxide nanocatalysts (Nanostructured & Amorphous Materials, Houston, TX, USA) with an average particle size of 21.6 nm. Salt precursors (FeCl_3_∙6H_2_O, NiCl_2_∙6H_2_O, CoCl_2_∙6H_2_O, and Pd(NO_3_)_2_∙2H_2_O) were supplied by Merck KGaA, Darmstadt, Germany. Each system was named considering the initials of the support, the elements with which the doping was made and the percentage of impregnation. For example, CeNi1Pd1 is a system supported on CeO_2_, with Ni and Pd at 1.0% (*w*/*w*). Details of nanoparticle characterization are found in our previous work [38]. Table 1 shows some properties of the employed nanocatalysts. 

The molar fraction for the couples Ni:Pd, Fe:Pd, and Co:Pd, are 0.4:0.7, 0.9:0.7, and 1.4:0.9, respectively. Considering the metal dispersion results, the nominal molar ratio affects the structure of nanocrystals of transition element (NOT)- Pd, causing a lower possibility of sintering processes for higher Pd/NOE molar value, (1.75 Pd/Ni, 0.78 Pd/Fe, and 0.64 Pd/Co).

X-ray photoelectron spectroscopy analysis was done to characterize the nanocatalysts’ surface chemistry following the protocol described in previous work [42]. The atomic content and relationships from O1s, Ce3d, and Pd3d profiles are shown in Table 2. The O1s pattern was deconvoluted in two main peaks at 529.0 eV and 531.5 eV ascribed to lattice oxygen (O_latt_) and ascribed to the surface (O_ads_) [43]. Between the functionalized nanocatalysts, the amount of O_ads_ follows the increasing order CeCo1Pd1 < CeFe1Pd1 < CeNi1Pd1, therefore, O_ads_/O_latt_ follows the same order. 

The peaks for Ce3d_5/2_ and 3d_3/2_ that appear at 881.6 eV, 885.3 eV, 899.8 eV, and 903.0 eV, which are associated to Ce^3+^ species. In the case of Ce^4+^ it is found at 882.7 eV, 889.7 eV, 898.6 eV, 901.3 eV, 907.6 eV, and 919.3 eV [44]. The increasing sequence for Ce^3+^ is CeCo1Pd1 < CeFe1Pd1 < CeNi1Pd1, which agrees with the sequences of O_ads_ and O_ads_/O_latt_. The formation of Ce^3+^ is intrinsically related to the oxygen vacancies formation, promoting the activation of surface oxygen. 

From Pd3d pattern, four main peaks are found. The binding energy at 337.2 eV (Pd 3d_5/2_) and 344.5 eV (Pd 3d_3/2_) refers to Pd^2+^ in bulk PdO [45]. The content of Pd^2+^ increases in the same order than Ce^3+^, O_ads_, and O_ads_/O_lat_, demonstrating the correlation between the three species. In the case of Pd^2^^+^, they can function as a reservoir for oxygen adsorption. The other peak at 335.2 eV and its respective satellite refer to the metallic palladium, which follows the opposite behavior than Pd^2^^+^.

A Colombian extra-heavy crude oil (EHO) of 6.4° API, viscosity of 3.1 × 10^6^ cP at 25 °C was used for isolation of *n*-C_7_ asphaltenes (A) and resins II (R). The content of asphaltenes and total resins were 12.64 and 51.98 wt%, respectively. *n*-Heptane (99%, Sigma-Aldrich, St. Louis, MO, USA) was used to isolate the asphaltenes from the EHO, and chromatographic silica (Sigma-Aldrich, St. Louis, MO, USA) was added to the de-asphalted crude oil to separate the resins from the solution. Both fractions were isolated following the protocols described elsewhere [46,47]. Finally, toluene (99%, Sigma-Aldrich, St. Louis, MO, USA) was used as a solvent for the adsorption experiments.

### 2.2. Methods

#### 2.2.1. Asphaltenes and Resins Characterization

The characterization of individual *n*-C_7_ asphaltenes and resins II was carried out through elemental analysis (C, H, N, S, and O) following the ASTM D5291 standard method [48] using an elemental analyzer (Perkin-Elmer, Waltham, MA, USA). Average molecular weight ($M_{W}$) was obtained by vapor pressure osmometry (VPO) using a Knauer osmometer (Knauer, Berlin/Heidelberg, Germany). ^1^H NMR and ^13^C NMR were carried out in a Bruker AMX 300 spectrometer (Karlsruhe, Germany). X-ray photoelectron spectrometry analysis was obtained in a PHOIBOS 150 1D-DLD analyzer (SPECS, Berlin, Germany). The procedures are described elsewhere [49]. Based on these results, average representative molecules for both fractions were constructed and optimized by density functional theory applying Becke’s three-parameter and Lee-Yang-Parr functions. Material Studio (BIOVIA, San Diego, CA, USA) was used for this purpose. 

#### 2.2.2. Adsorption Experiments 

Adsorption isotherms were constructed at 25 °C by making stock solutions of 2.2 mmol·L^−1^ of resins II and/or *n*-C_7_ asphaltenes in toluene and 100 mg of nanocatalysts per 10 mL of volume solution. Initial concentrations of resins II were varied between 0.1 mmol·L^−1^ and 2.2 mmol·L^−1^, and asphaltenes between 0.1 mmol·L^−1^ and 1.5 mmol·L^−1^. The instrument and protocol employed for the adsorption isotherms construction were like those described in the previous studies [23,43,50,51].

In the first stage, the individual components’ adsorption was performed in the three bielemental systems (i.e., CeNi1Pd1, CeCo1Pd1, and CeFe1Pd1) and the support CeO_2_. The system with higher adsorption was then selected to evaluate the competitive adsorption in different R:A mass ratios of 8:2, 1:1, and 2:8. The amount adsorbed of each component was obtained by combining softening point (SP), and thermogravimetric (TGA) experiments following the ASTM E28-12 standard methods [50]. Figure S1 shows the calibration curve of SP against *n*-C_7_ asphaltenes percentage where a R^2^ value close to the unity was obtained. 

Finally, this work uses the Solid–Liquid Equilibrium (SLE) model to describe the adsorption isotherms based on the theory of adsorption and association of molecules on microporous surfaces [51]. The model is described in Section S.1 of the Supplementary Materials.

#### 2.2.3. Steam Catalytic Gasification of Resins II and *n*-C_7_ Asphaltenes

The steam catalytic gasification of virgin and adsorbed compounds on nanocatalysts was performed using a Q50 thermogravimetric analyzer (TA Instruments, Inc., New Castel, DE, USA). Two different processes were executed. The first one, under non-isothermal heating between 100–600 °C, at a heating rate of 20 °C·min^−1^. In the second one, the samples were subjected to isothermal heating at three different temperatures. Samples in the absence of nanocatalysts were heated at 370 °C, while the asphaltenes/resins-containing nanocatalysts at 220 °C. The steam atmosphere was simulated by introducing 100 mL·min^−1^ of N_2_ and 6.30 mL·min^−1^ of H_2_O_(g)_ using a gas saturator controlled by a thermostatic bath at atmospheric pressure [37].

Nanocatalysts with the highest catalytic activity were selected to evaluate the isothermal conversion of different R:A ratios. Catalytic experiments were developed for an adsorbed asphaltene and/or resin II amount of 0.0002 mmol·m^−2^ ± 0.00002 mmol·m^−2^ [52]. The experimental results were used to calculate the activation energy according to the isothermal procedure [53] described in Section S.2 of the supplementary material. 

Gas monitoring during the experiments was done in a mass spectrometer (Shimadzu GC-MS, Tokyo, Japan) coupled to the TGA. The equipment was adjusted at an ion trap linear scan rate of 0.03 m/z between 0 and 200 m/z and an electron impact mode of 100 eV to obtain detailed information of the sample’s gasification. The gases were analyzed following the protocols described elsewhere [24,49,54,55]. The evolved gases during steam catalytic gasification were evaluated at three different temperatures (210 °C, 220 °C, and 230 °C). Each run was repeated, at least twice, to be ensured of the reproducibility of the experiments.

## 3. Results

### 3.1. Resins II and n-C_7_ Asphaltenes Characterization

The estimated elemental composition of *n*-C_7_ asphaltenes and resins II is shown in Table 3. The resins I data was also reported in Table 3 taken from our previous work for comparative purposes only [56]. The content of heteroatoms differs for each fraction. Nitrogen was lower than 0.5% for *n*-C_7_ asphaltenes and resins II, while sulfur content was similar for resin II and *n*-C_7_ asphaltene. As expected, the most significant difference between the two resins is the oxygen content, which was higher for resins II than that for resins I. Due to the strong aromaticity of *n*-C_7_ asphaltenes and their degree of unsaturation, resins II and I have a higher H/C ratio. The average molecular weight follows the increasing order resin I < *n*-C_7_ asphaltenes < resin II. This result shows that resin II isolated from the EHO employed in this study is heavier than asphaltenes.

Table 4 and Table 5 show the XPS and NMR results for both fractions, respectively. The survey spectra for both fractions indicate X-ray induced Auger transitions of oxygen, nitrogen, carbon, and sulfur. Analyzing the high-resolution spectra, the following results were obtained: Oxygen was present as single bonds (C–O–C, C–O) (532.4 eV) or carboxyl (533.5 eV) in both fractions. Carboxyl groups were in a higher percentage in resins II than *n*-C_7_ asphaltenes. The C1s was divided into single bonds (C–C, C–H) (284.6 eV), C=O (287.9 eV), and COO– (289.4 eV) species. As expected, C–C and C-H predominate for the both samples, while C=O was higher for *n*-C_7_ asphaltenes and COO– for resins II. Although nitrogen was present in low concentration for *n*-C_7_ asphaltenes (elemental analysis), it was slightly detected by XPS. The spectra were deconvoluted in three peaks (i.e., pyridinic (398.8 eV), pyrrolic (399.9), and amines (400.7 eV)) for both fractions. The pyridinic content was the highest for both and the lowest for amines. Finally, sulfur high-resolution spectra show the presence of thioethers (163.4 eV), thiophenes (164.4 eV), and sulfones (167.7 eV). For asphaltenes, the sulfur form percentage increases in the order sulfones (1.8%) < thioethers < (23.4%) < thiophenes (74.8%). In resins the trend changes as follows: thiophenes (25.2%) < sulfones (25.3%) < thioethers (49.5%).

Results from NMR shows that hydrogen was found in four different forms: Aromatic hydrogen (H_a_), and aliphatic hydrogens α, β, and γ linked to aromatic rings, (H_α_, H_β_, and H_γ_, respectively). H_a_ content was higher for *n*-C_7_ asphaltenes than resins II. For both fractions, the H_β_ species were in higher proportion than H_α_ and H_γ_. This result indicates the predominant presence of paraffinic/naphthenic β-hydrogens in aliphatic and aromatic structures, respectively. Resins II present a higher content of long aliphatic chains in γ-position (H_γ_) than *n*-C_7_ asphaltenes [57]. From the ^13^C NMR spectra, carbon was found in aromatic (C_ar_) and aliphatic (C_al_) form. As expected, resins II present a predominant C_al_ content (60.96%) and asphaltenes a higher proportion of C_ar_ (64.48%). 

**Figure 1:**
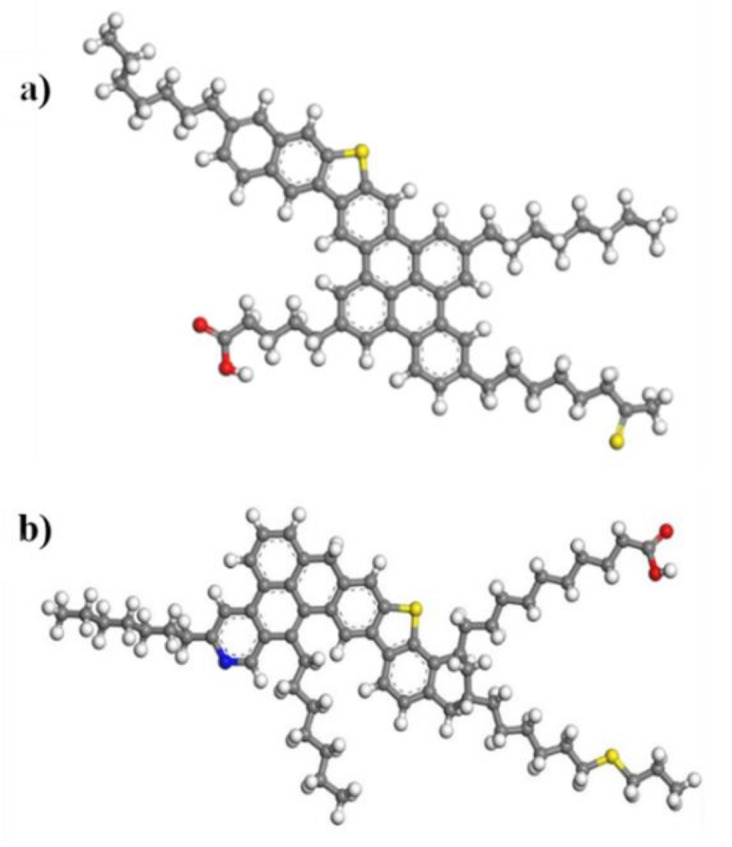
Average (**a**) *n*-C_7_ asphaltene and (**b**) resins II molecule model constructed from DFT analysis. The carbon, hydrogen, oxygen, nitrogen, and sulfur are represented by the green, white, red, blue, and yellow atoms, respectively.

Based on the general properties obtained from elemental analysis, molecular mass, nuclear magnetic resonance, and X-ray photoelectron spectroscopy, average structure molecules were proposed. Figure 1 shows the *n*-C_7_ asphaltene and resin II molecules. The samples show the continental type model widely accepted for both fractions. *n*-C_7_ asphaltene molecular formula proposed is C_62_H_70_S_2_O_2_, the theoretical molecular weight of 911.36 g·mol^−1^ and deviation of 0.4%. Resin II molecular formula proposed is C_63_H_85_NS_2_O_2_, molecular weight estimated in 952.5 g·mol^−1^ and deviation of 0.5%. 

### 3.2. Adsorption Isotherms

**Figure 2:**
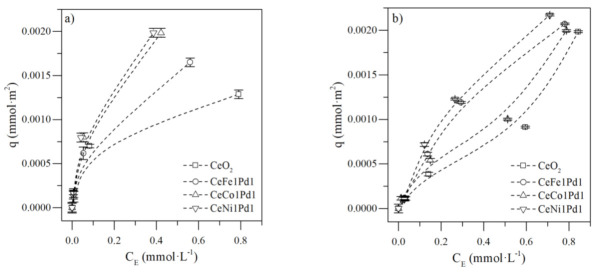
Adsorption isotherms of (**a**) *n*-C_7_ asphaltenes and (**b**) resins II onto CeNi1Pd1, CeFe1Pd1, CeCo1Pd1, and CeO_2_ nanocatalysts evaluated at 25 °C. The solid lines represent the prediction of the SLE model and the symbols are experimental data. Panel (**a**) was taken from previous work [38].

Figure 2 shows the adsorption isotherms of *n*-C_7_ asphaltenes (panel a) and resins II (panel b) onto CeNi1Pd1, CeCo1Pd1, CeFe1Pd1, and CeO_2_ nanocatalysts. A high affinity observed between the different nanocatalysts and the *n*-C_7_ asphaltenes is related to the type Ib isotherms following the International Union of Pure and Applied Chemistry (IUPAC) [58].

Adsorption capacity at any concentration decreases in the order CeNi1Pd1 > CeCo1Pd1 > CeFe1Pd1 > CeO_2_ for *n*-C_7_ asphaltenes; whereas, for resins II uptake, the order increases as: CeNi1Pd1 > CeFe1Pd1 > CeCo1Pd1 > CeO_2_. This result indicates an increase in the adsorptive capacity by NOT impregnation following the order Fe < Co < Ni for asphaltene adsorption, since the effective nuclear charge increases in the same order changing Lewis acidity [59]; hence, facilitating the formation of coordinated bonds between heteroatoms (Lewis bases) and metals of heavy hydrocarbons, and the active site of NOTs in the different nanocatalysts [60]. On the other hand, the resins II chemical structure changes regarding asphaltenes increase their selectivity for Fe crystals than Co.

According to the slope in Henry’s region for the adsorption isotherms curves of resins II on CeNi1Pd1 and CeFe1Pd1 nanocatalysts, a high affinity is observed. The isotherms obtained follow the IUPAC standard corresponding to type Ib isotherm [58], indicating that resins II have greater selectivity for the Ni and Fe functional groups [61]. Authors explain that aromatic nitrogen and sulfur species’ content has a high affinity for iron and nickel nanocatalysts [62]. Resins II adsorption on non-functionalized support and bielemental CeCo1Pd1 nanocatalyst shows an isotherm type II. At low concentrations, adsorption at the active sites accessible for resins II is high, indicating a high slope in Henry’s region; however, as the concentration of resins II increases, the formation of aggregates increases, generating adsorption multilayer and taking a profile of type II isotherms [63]. 

Comparing the adsorptive capacity of different samples, for a fixed concentration of resins, the adsorption amount increases in the following order CeO_2_ < CeCo1Pd1 < CeFe1Pd1 <CeNi1Pd1. Unlike the adsorption of *n*-C_7_ asphaltenes, the adsorption of resins II is greater for the Fe-Pd couple than that for the Co-Pd couple. Based on the results, the competitive adsorption in R:A systems was evaluated in CeNi1Pd1 nanocatalysts and Figure S2 shows the obtained results. 

Figure S2a shows that *n*-C_7_ asphaltene adsorption capacity on CeNi1Pd1 nanocatalysts decreases with resin II content. For 2:8 and 1:1 ratios, the isotherms obtained are classified as type I, while the isotherm for 2:8 system behaves type III according to the IUPAC [58]. The change in the asphaltene adsorption mechanism can be associated with a change in their colloidal structure or competitive adsorption between the two polar fractions. As the amount of resins II increases in the system, there is a reduction of the active surface sites available for *n*-C_7_ asphaltene adsorption. For example, at fixed C_E_ = 0.33 mmol·L^−1^, the asphaltene adsorption amount was 0.002 mmol·m^−2^, 0.0018 mmol·m^−2^, 0.0011 mmol·m^−2^, and 0.0008 mmol·m^−2^ for *n*-C_7_ asphaltene, 2:8; 1:1, and 8:2 systems, respectively. 

**Figure 3:**
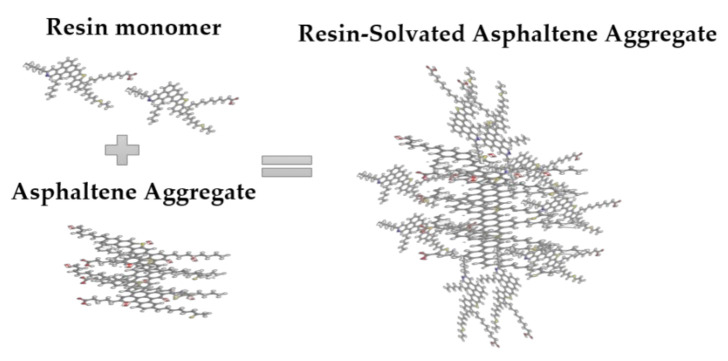
Interactions between resins and asphaltene aggregates. Own source.

Structurally, given the similarity between both fractions, it is likely that strong and stable interactions will occur between them. As shown in Table 1, asphaltenes and resins contain similar percentages of N-, S-. and O- heteroatoms. Because asphaltenes could interact with traces of resins containing oxygen and/or nitrogen, the primary interaction is likely of the attractive type. In this sense, resins tend to interact more with asphaltenes, as shown in Figure 3 below. 

When the amount of resins in the system is high enough, a solvation phenomenon could be generated, which reduces the asphaltene–nanoparticle interactions by blocking important active sites, such as aromatic, naphthenic rings, and heteroatoms.

Figure S2b shows the adsorption isotherms for resins II at different R:A ratios. The results show that type I isotherms for 8:2 and 1:1 ratio. However, in 2:8 system, the isotherm obtained is changed to type III. Results suggest that resins II adsorption is affected by *n*-C_7_ asphaltene content. The multilayer behavior of 2:8 ratio show that the resin-asphaltene interactions prevail over resin–resin interactions [64]. The adsorption amount of resins at C_E_ = 0.33 mmol·L^−1^ were 0.0013 mmol·m^−2^, 0.0011 mmol·m^−2^, 0.0007 mmol·m^−2^, and 0.0005 mmol·m^−2^ for individual resins, 2:8; 1:1, and 8:2 systems, respectively.

Table S1 summarizes H, K, y $Q_{m}$ values of the SLE model for each system evaluated. The experimental data adjustment through the SLE model shows the highest affinity of both resin II and *n*-C_7_ asphaltene fractions for CeNi1Pd1 nanocatalysts according to the low H values. Resins II presented lower K values than *n*-C_7_ asphaltenes, which could result from the lower amount of C_ar_ on their structure. It is reported that the aggregation mechanism of both fractions occurs through π-π stacking interactions between the aromatic cores. Hence, the lower C_ar_ the lower the self-association degree on the nanocatalysts surface. On the contrary, the H values were higher for *n*-C_7_ asphaltenes than those for resins II due to the higher content of carboxyl, thiophene, and pyrrolic functional groups. It is worth mentioning that resins presented a higher value for Fe-Pd couple due to the high content of pyridines, which present a high affinity for this NOT.

Concerning the self-association degree of *n*-C_7_ asphaltenes and resins, each fraction’s parameter decreases as the content of the other fraction increases. This result suggests that asphaltene–asphaltene and resins–resins interactions are affected by stronger resin-asphaltene interactions [64,65]. In this case, multilayer adsorption of *n*-C_7_ asphaltenes on CeNi1Pd1 nanocatalysts is favored for higher R:A ratios. 

To understand the adsorption mechanism of *n*-C_7_ asphaltenes in resins II presence on nanoparticles, the adsorbed and desorbed amount of asphaltenes was predicted. If the colloidal state of *n*-C_7_ asphaltenes is affected by resin II, the predicted and the experimental adsorbed amount will differ. The predicted adsorbed amount in R:A systems is calculated considering as a fraction of the individual adsorbed amount of each component. For example, in 8:2 system the predicted adsorbed amount of *n*-C_7_ asphaltenes are assumed as 20.0% of the individual mass fraction adsorbed. A linear graph $q_{predicted}$ against $q_{experimental}$ with a slope m = 1, intersection b = 0, and $R^{2}$ = 1.0 is associated with a good prediction. Table S2 shows the results for the predicted adsorbed amount of *n*-C_7_ asphaltenes on CeNi1Pd1 nanoparticles in R:A systems. The results show a good fit between the predicted and experimental values. The values of m were close to 1 in all cases, and b were close to the origin. These results give a clear idea about the influence of resins II on the phenomenon evaluated, indicating that the adsorption of *n*-C_7_ asphaltenes are mainly controlled by their concentration in the systems and by the affinity of the nanoparticle for resins II. In this way, the colloidal state of the asphaltenes is not affected by the presence of resins II. These results are in a good agreement with the reported results in previous works [18,66].

### 3.3. Thermogravimetric Experiments.

**Figure 4:**
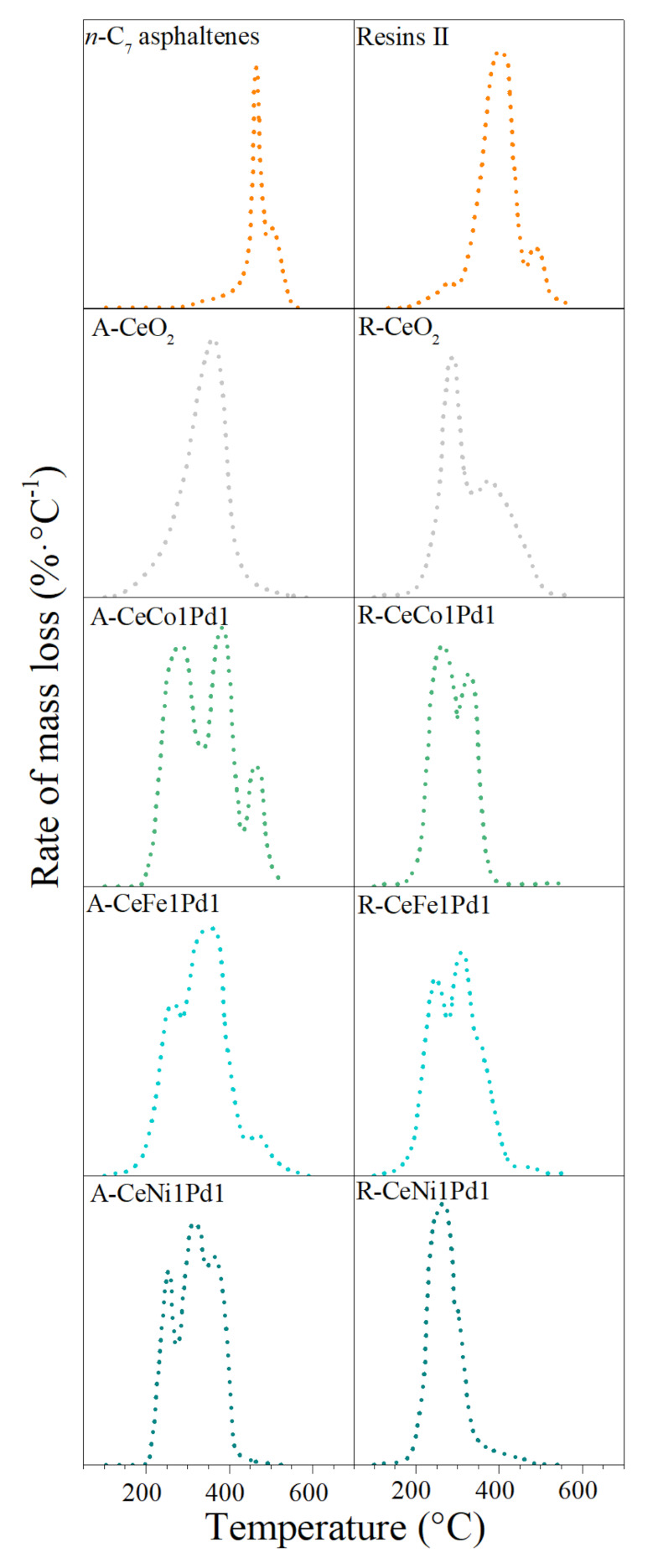
Rate of mass loss for *n*-C_7_ asphaltenes and resins II steam gasification in the absence and presence of CeO_2_, CeNi1Pd1, CeCo1Pd1 and CeFe1Pd1 nanocatalysts. N_2_ flow rate = 100 mL·min^−1^, H_2_O_(g)_ flow rate = 6.30 mL·min^−1^, heating rate = 20 °C·min^−1^, asphaltene load = 0.0002 mmol·m^−2^, resins load = 0.0002 mmolg·m^−2^. Thermograms for *n*-C_7_ asphaltenes were taken from Medina et al. [38].

Figure 4 shows the rate for mass loss for the steam catalytic gasification of *n*-C_7_ asphaltenes and resins II, respectively. Results reveal that pure resins II and *n*-C_7_ asphaltenes decompose around 420 °C and 450 °C, respectively (rate for mass loss peak). However, with nanocatalysts, the decomposition temperature reduces, and decomposition begins at approximately 200 °C in all systems. 

Bielemental and support systems reduce the *n*-C_7_ asphaltenes decomposition temperature from 450 °C, to 220 °C, 230 °C, 250 °C, and 370 °C for CeNi1Pd1, CeCo1Pd1, CeCo1Pd1, and CeO_2,_ respectively. On the other hand, the three bielemental and CeO_2_ nanocatalysts decompose resins II at 220 °C and 300 °C, respectively. The conversion of both fractions continues at high temperatures around 350 °C, as a result of the distribution of high, medium, and low molecular weight hydrocarbons [29].

The decomposition temperature peaks of resins II suggest a high content of lower molecular weight hydrocarbons (aliphatic structures) than those present in the asphaltenes’ molecular structure. This agrees with ^13^C-NMR results. In both cases, nanocatalysts’ catalytic activity was improved with the addition of NOT in the order of Fe < Co < Ni. The highest performance for the couple Ni-Pd is due to the support’s ability to interact with Ni and Pd nanocrystals to promote cracking and isomerization reactions [45,67,68,69]. The strong metal-support interaction between Ce^3+^ ions and Ni nanocrystals may promote water gas shift reactions at low temperatures [67]. During this process, the support conducts a redox cycle, which generates oxygen anion vacancies ($V_{o}^{2•}$). These are active sites for the adsorption of steam molecules and, therefore, greater interaction with the adsorbed heavy oil fractions [45,68].

**Figure 5:**
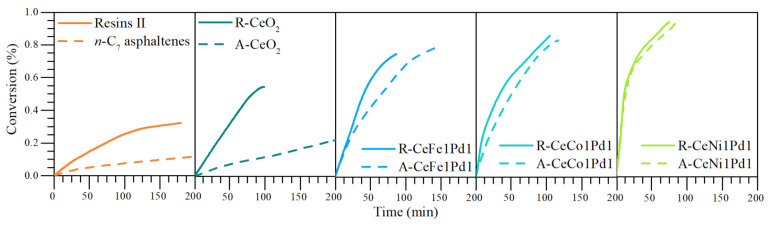
Isothermal conversion for *n*-C_7_ asphaltenes and resins II in the absence (370 °C) and presence of CeNi1Pd1, CeFe1Pd1, CeCo1Pd1, and CeO_2_ nanocatalysts (220 °C). N_2_ flow rate = 100 mL·min^−1^, H_2_O_(g)_ flow rate = 6.30 mL·min^−1^, asphaltene load = 0.0002 mmol·m^−2^, resin load = 0.0002 mmol·m^−2^. Isothermal conversions of *n*-C_7_ asphaltenes were taken from Medina et al. [38].

Figure 5 shows the isothermal conversion at 220 °C for *n*-C_7_ asphaltenes and resins II adsorbed on the bielemental systems and the support. It is appreciated that the conversion degree increases in the order of CeO_2_ < CeFe1Pd1 < CeCo1Pd1 < CeNi1Pd1 in both fractions.

Between the support and the best bielemental nanoparticle (Ni-Pd), to convert 20% of resins II and *n*-C_7_ asphaltenes, the time decreases approximately 70 min and 155 min, respectively. Among many factors, this is due to the role played by the Ni-Pd couple. NiO nanocrystals can inhibit PdO nanocrystals growth [38]; therefore, they acquire a higher dispersion than Co-Pd and Fe-Pd bielemental systems, generating many HA-PdO interactions. This implies an increase in hydrogen and light gas production capable of stabilizing the cracked heavy oil fractions’ free radicals [7].

**Figure 6:**
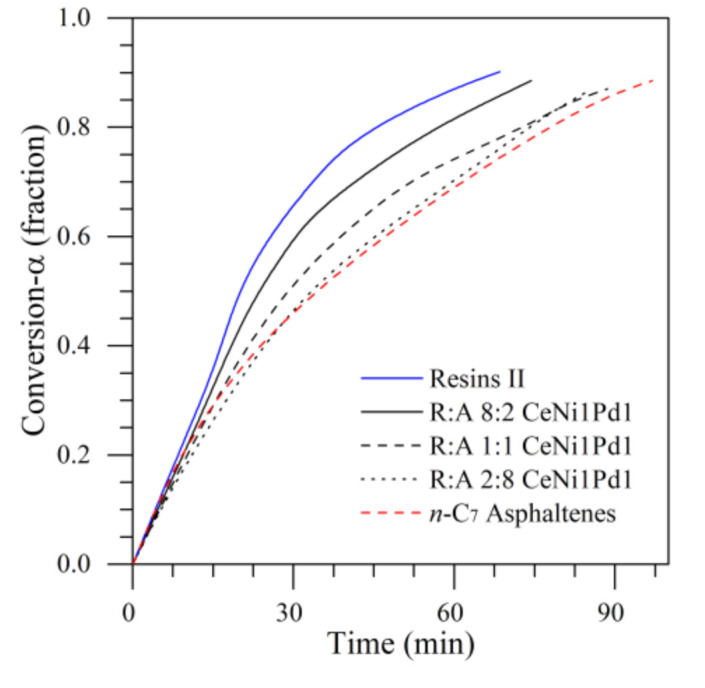
Isothermal conversion for different R:A ratios adsorbed on CeNi1Pd1 nanocatalysts at 220 °C. N_2_ flow rate = 100 mL·min^−1^, H_2_O_(g)_ flow rate = 6.30 mL·min^−1^, asphaltene/resin load = 0.0002 mmol·m^−2^.

Figure 6 shows the isothermal conversions at 220 °C for R:A ratios of 8:2, 1:1, and 2:8 on CeNi1Pd1 nanocatalysts. The conversion degree increases in the order A < 2:8 < 1:1 < 8:2 < R at any time. It was reported that resins are composed for a higher degree of aliphatic carbon structures. This composition influences the number of interactions generated in R:A systems between asphaltenes and resins, and therefore on catalysis [56]. Decomposing structures by R–R bonds is less complicated than breaking R-A structures [56]. Additionally, a higher asphaltene content, the presence of thiophenes and pyrroles prevails; therefore, more time is required for their cracking [49]. In addition, the first cracked products can polymerize and form coke.

**Figure 7:**
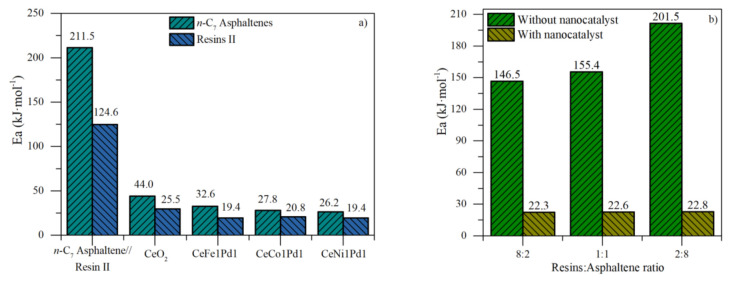
Estimated effective activation energy for isothermal gasification of (**a**) *n*-C_7_ asphaltenes and resins II with CeO_2_, CeFe1Pd1, CeCo1Pd1 and CeNi1Pd1 nanocatalysts, and (**b**) R:A systems with and without CeNi1Pd1 nanocatalysts.

The effective activation energy was used to estimate the energy requirements needed to carry out the chemical reaction. Figure 7a shows the results of effective activation energy ($E_{a}$) for *n*-C_7_ asphaltenes and resins II with and without bielemental nanocatalysts and support. $E_{a}$ decreases considerably by the addition of nanocatalysts, in the order of CeO_2_ < CeFe1Pd1 < CeCo1Pd1 < CeNi1Pd1. NOTs impregnation modifies the decomposition mechanism of the heavy oil fractions, indicating that the catalyst’s chemical nature influences gasification reactions. On the other hand, the energy required for thermo-gasification and thermo-catalytic gasification is lower for resins II than that for asphaltenes. In addition, the estimated activation energies values for the R:A systems above CeNi1Pd1 nanocatalysts are shown in panel b. It is observed that $E_{a}$ increases in the order of 8:2 < 1:1 < 2:8 for virgin and adsorbed fractions. CeNi1Pd1 nanocatalysts reduced the activation energy from 146.5, 155.4, and 201.5 kJ·mol^−1^ to 22.3, 22.6, and 22.8 kJ·mol^−1^, for 8:2, 1:1 and 2:8 systems, respectively.

### 3.4. Analysis of Gaseous Profiles during Steam Gasification

#### 3.4.1. Effect of Nanocatalyst Chemical Nature

**Figure 8:**
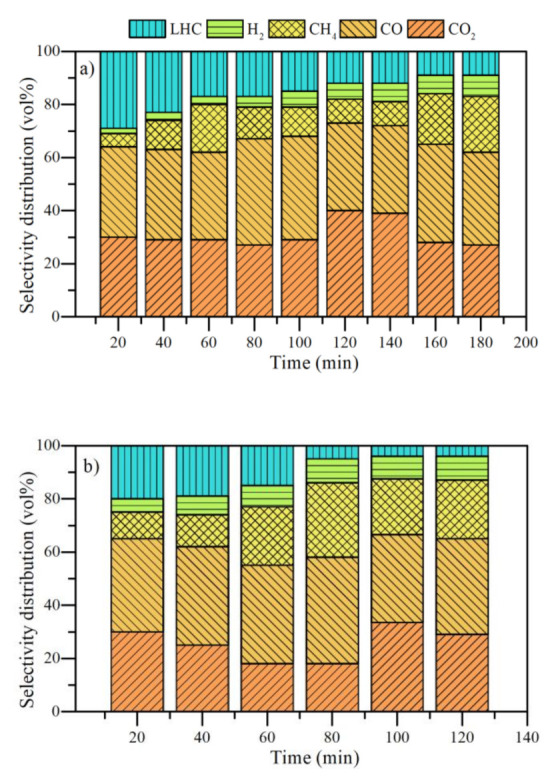
Selectivity distribution of light gases produced from the isothermal conversion of (**a**) *n*-C_7_ asphaltenes and (**b**) resins at 370 °C. N_2_ flow rate = 100 mL·min^−1^, H_2_O_(g)_ flow rate = 6.30 mL·min^−1^.

The effect of nanocatalyst chemical nature on evolved gases during the steam gasification of *n*-C_7_ asphaltenes and resins II was analyzed under isothermal heating at 220 °C and the results are shown in Figure 8 as a function of time. It is observed that the release of CO_2_, CH_4_, CO, a mixture of light hydrocarbons (LHC) and H_2_. Some traces of other gases were evidenced in different time intervals, but their contribution was less than 0.1%; thus, they were neglected. 

It is worth mentioning that non-catalyzed systems were evaluated at 370 °C due to their complex structure. The gasification results for the non-catalyzed systems show considerable CO and CO_2_ production during the conversion of the samples. The higher production of CO is associated with the reverse Boudouard reaction. As the reaction is endothermic, the production of CO becomes meaningful over time. On the other hand, the LHC mixture was composed mainly of C_2_H_2_, C_2_H_4_, and C_6_H_6_. Its production was markedly at the beginning of the warm-up. This is because the aliphatic chains break down at low temperatures and promote the production of these gases. However, over time, both asphaltenes and resins produce a higher proportion of CO_2_ and CO and do not promote intermediate reactions that give rise to LHC.

On the other hand, methane and hydrogen were produced in low quantities all the time evaluated. It should be noted that these fractions decompose less than 20% of their mass at 370 °C during the time analyzed. It is noted that asphaltenes produce a higher amount of CO_2_ and a lower amount of H_2_ than resins II in all the time range evaluated. This result agrees with the higher content of aromatic carbon in asphaltenes. During thermal heating, the aromatic core is susceptible to the formation of ketones and finally releasing as CO_2_. The higher the content of hydrogens α and γ linked to aromatic rings, the higher the hydrogen release. 

**Figure 9:**
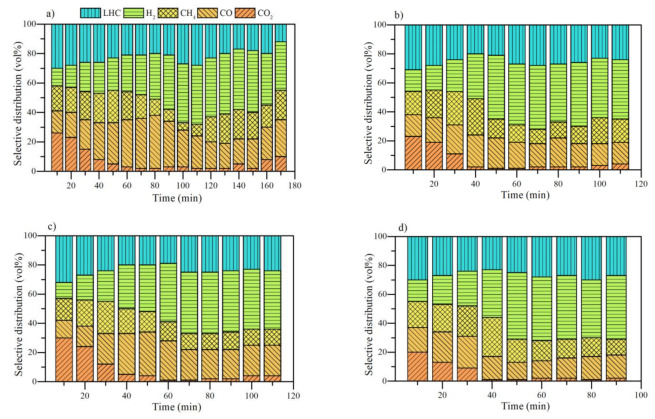
Selectivity distribution of light gases produced from the isothermal conversion of *n*-C_7_ asphaltenes at 220 °C in the presence of (**a**) CeO_2_, (**b**) CeCo1Pd1, (**c**) CeFe1Pd1, and (**d**) CeNi1Pd1 nanocatalysts. N_2_ flow rate = 100 mL·min^−1^, H_2_O_(g)_ flow rate = 6.30 mL·min^−1^, asphaltene load = 0.0002 mmol·m^−2^.

**Figure 10:**
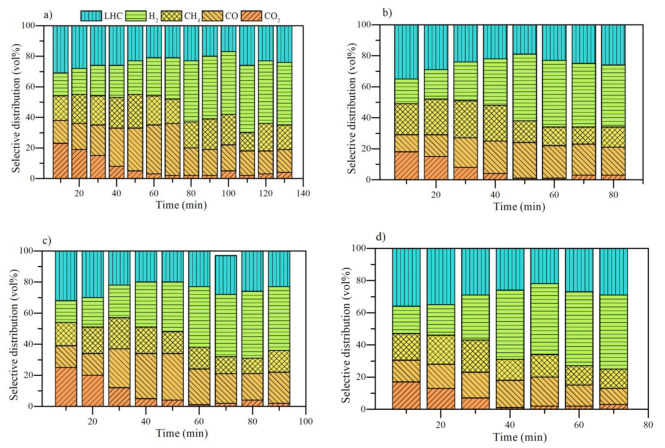
Selectivity distribution of light gases produced from the isothermal conversion of resins II at 220 °C in the presence of (**a**) CeO_2_, (**b**) CeCo1Pd1, (**c**) CeFe1Pd1, and (**d**) CeNi1Pd1 nanocatalysts. N_2_ flow rate = 100 mL·min^−1^, H_2_O_(g)_ flow rate = 6.30 mL·min^−1^, resin load = 0.0002 mmol·m^−2^.

Figure 9 and Figure 10 show the selectivity distribution of light gases for *n*-C_7_ asphaltenes and resins adsorbed on different nanocatalysts, respectively. The results reveal an increase in LHC, CH_4_, and H_2_ in all the times at 220 °C for both fractions. The asphaltenes begin their decomposition with a CO_2_ production between 20% and 30% (vol) for all nanocatalysts. Unlike the virgin fractions, there is a higher production of H_2_ and LHC from the first 10 min of heating. Subsequently, CO_2_ production falls to a greater degree for asphaltenes adsorbed on CeNi1Pd1 than in the rest of the systems. In turn, the production of CO, CH_4_ and H_2_ increases. When reacting with CO_2_, carbon produces CO, while CH_2_ is produced by breaking short aliphatic chains (methyl, methylene, etc.). Hydrogen in the first instance can be produced by the reaction of C in asphaltenes and H_2_O_(g)_ molecules.

During the thermal gasification there is a drop in the CH_4_ production in all cases. For CeO_2_ it occurs at 50 min, for CeFe1Pd1 at 40 min, and for CeCo1Pd1 and CeNi1Pd1 at 30 min. In turn, the production of H_2_ increases. This may suggest the development of steam reforming reactions assisted by different nanocatalysts [69]. The difference in the time in which occurs, is a response to each system’s catalytic activity. Therefore, the most catalytic system (CeNi1Pd1) generates this change in less time and with more outstanding vol% production of H_2_. Likewise, the drop in CO and a slight increase in CO_2_ and H_2_ indicate the WGS reaction development [69]. These changes in the selective distribution of gaseous products were first observed in the system (CeNi1Pd1) between 40 and 50 min. Moreover, the increase in CO_2_ was minimal. The system that took the longest was CeO_2_ (90–100 min). Regarding several studies on the catalytic gasification of carbon, all the materials in this study produced a lower content of CO_2_ in the entire time interval analyzed [5,70,71,72].

Finally, the mixture of light hydrocarbons was mainly composed of C_2_H_2_, obtaining a total gas mixture with an average calorific value of 82.45 kcal·kg^−1^ for CeO_2_, 102.51 kcal·kg^−1^ for CeFe1Pd, 124.23 kcal·kg^−1^ for CeCo1Pd1, and 139.14 kcal·kg^−1^ for CeNi1Pd1 on time evaluated. It is observed that the dispersion and average crystallite size of Pd in functionalized systems follows the same order as the increase in H_2_ release and the average calorific values of the release gas mixture. As higher the dispersion and lower the average crystal size, the higher the calorific values and the content of H_2_ produced.

Figure 10 depicts the results for resins II. The resins followed the same trend as *n*-C_7_ asphaltenes. The main differences lie in how the nanocatalysts generate significant changes in the selective distribution of gases and their volumetric proportion.

The maximum production of CO_2_ in all cases is observed at 10 min, which follows the incremental order of CeNi1Pd1 < CeFe1Pd1 < CeCo1Pd1 < CeO_2_. The order in which the LHCs were produced in this interval follows the opposite order. This indicates that in the presence of the CeNi1Pd1 catalyst, the resins decompose a high content of their aliphatic structure. The production of CO_2_ tends to decrease, being more noticeable in the catalyst CeNi1Pd1. In other words, the catalyst promotes C-CO_2_ interactions, increasing the release of CO [69]. Methane reforming reactions appear to occur between 30 and 40 min for functionalized catalysts and between 60 and 70 min for CeO_2_ support. On the other hand, the production of H_2_ intensified with the reduction of CO; that is, there is a participation of this gas in WGS reactions. This reaction occurs at higher temperatures, between 40 and 60 min for CeNi1Pd1, 60 and 80 min for CeCo1Pd1 and CeFe1Pd1, and 90 and 100 min for CeO_2_.

The future trend in hydrogen production is increasing toward Fe < Co < Ni for both fractions. Many researchers cite the oxygen storage/reduction of cerium-based materials and nanocrystals’ catalytic activity on the supports’ surface as a key component of the WGS and methane reforming activity of catalysts [67,69,73,74,75]. 

There have also been studies showing that the addition of dopants or a combination of multiple metals as active phases creates defects in the structure and oxygen vacancies in which pure, highly dispersed metal catalysts are located [62,67]. This allows the adsorption of reactants and facilitates the WGS and methane reforming reactions themselves. The hydrogen production trend is also agreed with each material’s catalytic activity for decomposing both fractions at the evaluated conditions. CeNi1Pd1 showed a higher performance than the other three samples and led to a higher hydrogen vol% in the effluent gas.

Cerium nanoparticles as active support facilitate the production of low molecular weight compounds due to (a) the positive effect of the NOT and NON elements and ceria interactions, and (b) the activity of redox couple Ce^3+^/Ce^4+^. There is evidence that oxygen atoms migrate from the support (CeO_2_) to transition element particles at higher temperatures during spillover phenomena. This oxygen can be used to oxidize CO to CO_2_, and subsequently, the partially reduced ceria can be re-oxidized by water, completing the catalytic cycle. Moreover, the gases can be used to produce H_2_ during the WGS reaction. In this sense, the surface of the support will be composed of Ce^3+^ and Ce^4+^ ions. Ce^3+^ ions formed after reducing Pd-based catalysts play an essential role in activating CO and CO_2_ molecules at the ceria-transition element interface. 

The heterogeneous surface of the material promotes different types of reactions. For example, the Ni/CeO_2_ active sites first generate the total combustion of CH_4_ followed by H_2_O and CO reforming. For Pd/CeO_2_, the reaction follows a direct mechanism with H_2_, CO, CO_2_, and H_2_O’s simultaneous formation. These differences are explained due to the transfer of electrons between the element’s particles and the support. In a steam atmosphere, the CO oxidation is enhanced by OH∙ and O_2_ species’ reaction to the support’s basic sites, following the gasification mechanisms, increasing CO_2_ and H_2_ production. Methane activation occurs through the active surface of oxygen. The high electronic affinity with the coordinated surface of unsaturated oxygen (cus) generates a strong interaction with methane and light hydrocarbon molecules, allowing the activation of C-H bonds at low temperatures and their subsequent breakdown, finally desorbing as CO_2_ and H_2_O.

**Figure 11:**
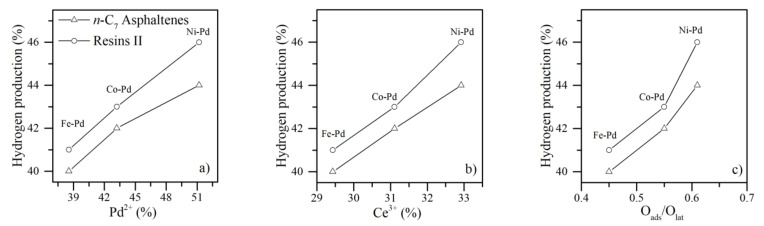
Hydrogen percentage produced from the isothermal conversion of *n*-C_7_ asphaltenes and resins II at 220 °C in the presence of functionalized nanocatalysts CeFe1Pd1, CeCo1Pd1, and CeNi1Pd1 as a function of the (**a**) Pd^2+^, (**b**) Ce^3+^ and (**c**) O_ads_/O_lat_ content. Hydrogen release at 90 min and 70 min was taken for constructing asphaltene and resins graphs.

The surface chemistry of the nanocatalysts was correlated with the results of selective distribution in hydrogen releasing from asphaltene and resin decomposition. The results are shown in Figure 11. As described, hydrogen release increases in the order CeFe1Pd1 < CeCo1Pd1 < CeNi1Pd1, which agrees with the tendencies for Pd^2+^, Ce^3+^ and O_ads_/O_lat_ in the surface chemistry of the nanocatalysts. It is well recognized the redox property of CeO_2_ influences catalytic activity. The relative abundance of Ce^3+^ and O_ads_/O_lat_ suggests their participation inf hydrogen formation. First, the redox cycle allows the oxygen adsorption under oxidation conditions and its release under reduced conditions, as is shown in Equations (3) and (4):

(3) CeO2−δ+ 12δ O2⇌CeO2

(4) CeO2⇒ CeCex + 2OOx ⇌CeCe·+ 12VO″+ 12 O2

Furthermore, hydroxyl groups (OH) formed by the reaction between H_2_O and partially reduced ceria oxygen vacancies react with CO to form methanoate species of bridge [76]. Above 170 °C, these species are transformed into bidentate ormats to decompose into final products of CO_2_ and H_2_ [76]. Similarly, the higher the content of Pd^2+^ species, the higher the selective distribution for hydrogen production in catalytic decomposition of both fractions. These ions could substitute Ce^4+^ in the ceria lattice to increase the defect concentration. Hence, the catalysts improved their oxygen vacancy density and oxygen mobility as increases Pd^2+^ in their surface. In complete agreement, it is reported that the strong interactions between Ni and Pd species with Ce support lead to more thermal stable metal species [38], which contributed to the synergistic effect of both phases for crude oil fractions decomposition and hydrogen production.

#### 3.4.2. Effect of R:A Ratio

**Figure 12:**
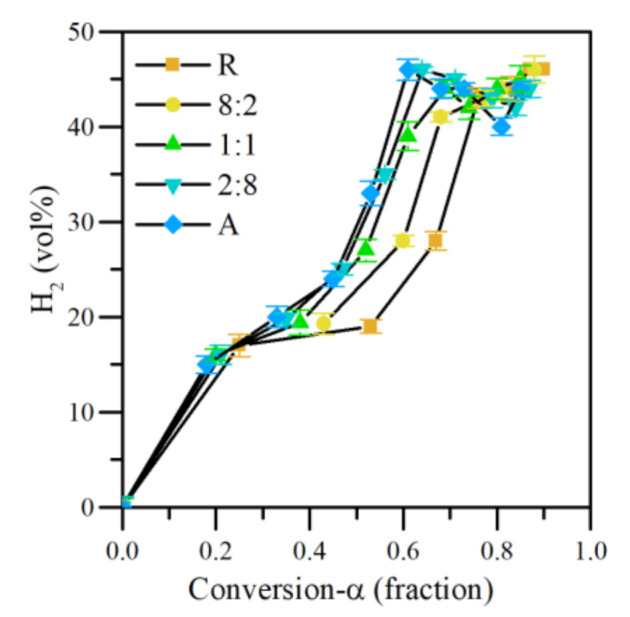
H_2_ production as a function of conversion degree of different R:A ratios adsorbed on CeNi1Pd1 nanocatalysts at 220 °C. N_2_ flow rate = 100 mL·min^−1^, H_2_O_(g)_ flow rate = 6.30 mL·min^−1^, asphaltene/resin load = 0.0002 mmol·m^−2^.

The CeNi1Pd1 was selected to evaluate the effect of R:A ratio on hydrogen release from its isothermal steam catalytic gasification at 220 °C. The results are shown as a function of the conversion degree (α) in Figure 12. It is found that the R:A ratio influences the H_2_ content on the effluent gas. At low conversions (α < 0.2) the H_2_ content is very similar in all systems. Between 0.4 and 0.7 the trend is evident. For the same conversion, the production of H_2_ increases in the order of R < 8:2 < 1:1 < 2:8 < A. As shown in the previous section, different reactions contribute to the generation of H_2_ in the presence of the CeNi1Pd1 catalyst. However, depending on the chemical nature of the adsorbed fraction, H_2_ release can vary. High content of short aliphatic chains promotes the production of methane in greater quantity, and as previously shown, asphaltenes have a higher content of H_α_ species. In this sense, methane reforming may have a greater contribution as the content of asphaltenes increases in R:A mixtures.

On the other hand, the systems show a production peak generated at lower conversions when the asphaltene content is lower. These data coincide with the slight increase in the production of CO_2_ in samples A and R, an indicator of WGS reactions. For conversions greater than 0.8, H_2_ production is fluctuating. However, it does not fall below 40.0 vol%. These results indicate the nanocatalyst’s potential to produce a hydrogen-rich gas from the steam catalytic gasification of asphaltenes and resins adsorbed both individually and in mixtures. All the results demonstrate the potential of the technology to release a hydrogen-rich gaseous mixture.

#### 3.4.3. Effect of Temperature

**Figure 13:**
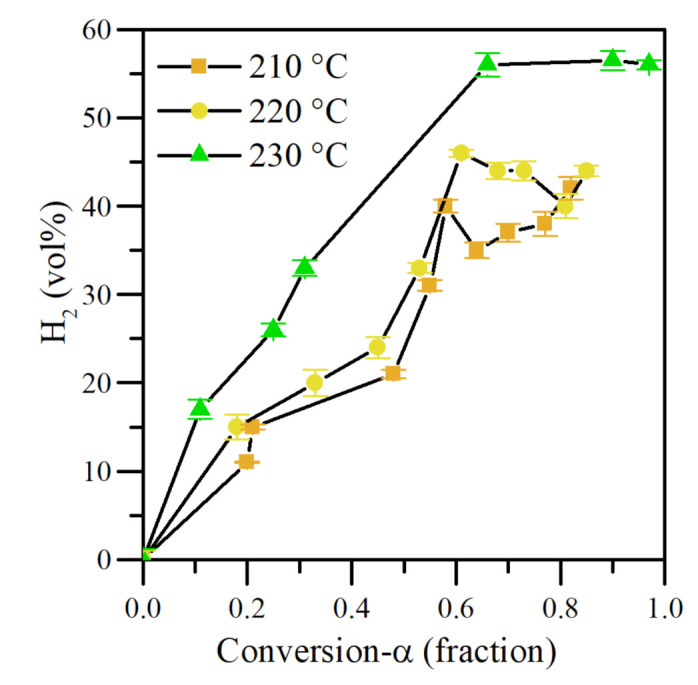
H_2_ production as a function of conversion degree of *n*-C_7_ asphaltenes adsorbed on CeNi1Pd1 nanocatalysts at 220 °C. N_2_ flow rate = 100 mL·min^−1^, H_2_O_(g)_ flow rate = 6.30 mL·min^−1^, asphaltene/resin load = 0.0002 mmol·m^−2^.

Operation temperature was varied between 210 and 230 °C to analyze its effect on H_2_ generation from the steam catalytic gasification of *n*-C_7_ asphaltenes. Figure 13 shows the results obtained. Higher H_2_ concentration was observed as the temperature increased. This indicates that at 230 °C the most favorable conditions for steam reforming and WGS reactions are received. This trend is following the degree of conversion of each fraction. In the same figure, it is observed that asphaltenes convert greater mass at 230 °C. Therefore, it produces a greater quantity of H_2_ catalyzed by CeNi1Pd1. The higher the temperature, the higher the H_2_O_(g)_ and CO_2_ gasification reaction rates, obtaining a higher release of H_2_ at 230 °C than the other two temperatures evaluated. 

## 4. Conclusions

For the first time, the present study shows the effect of resin II in the adsorption, and catalytic steam gasification of *n*-C_7_ asphaltene on hydrogen production using functionalized CeO_2_ nanocatalysts with different pairs of transition element oxides. Individual adsorption of resin II follows the order of Co < Fe < Ni. For the competitive adsorption, the simultaneous presence of two fractions influences the individual adsorption of *n*-C_7_ asphaltene and resin II correspondingly because when the amount of resin II increases on the system, the adsorption of *n*-C_7_ asphaltenes decreases. Additionally, resin II decomposition temperature decreased from 420 to 220 °C for bielemental nanocatalysts and 300 °C for the support of CeO_2_; whereas *n*-C_7_ asphaltenes decompose about 220 °C for bielemental nanocatalysts and 350 °C when the support catalyzes them. For the R:A systems, the conversion increases in the order of (R:A) 8:2 < 1:1 < 2:8, because asphaltenes have a greater content of heavy molecular weight components that require higher time for their decomposition. The effective activation energy is decreased by the presence of CeNi1Pd1 nanocatalysts in all R:A and individual systems, evidencing the positive thermal effect against the steam injection processes.

Hydrogen release was successfully quantified. The results depict an increasing H_2_ production order of CeO_2_ < CeFe1Pd1 < CeCo1Pd1 < CeNi1Pd1 under isothermal heating at 220 °C during asphaltene and resin conversion. The CeNi1Pd1 catalyst produces a gas effluent composed of more than 40 vol% of H_2_, decomposing both fractions. When the samples are heated at 230 °C, H_2_ production was increased close to 55 vol% during *n*-C_7_ asphaltene conversion. 

It was noted that asphaltenes produce a higher amount of CO_2_ and a lower amount of H_2_ than resins II in all the time range evaluated. This result agrees with the higher content of aromatic carbon in asphaltenes. During thermal heating, the aromatic core is susceptible to the formation of ketones and finally releasing as CO_2_. The higher the content of hydrogens α and γ linked to aromatic rings, the higher the hydrogen release. In the presence of CeNi1Pd1 a high efficiency to produce H_2_ was obtained regardless of the nature of the fraction and the R:A systems. That is, the decomposition of the R:A mixtures also generated gas contents similar to the individual fractions.

Likewise, CO_2_ production was minimal in all the cases evaluated, highlighting the use of the nanocatalysts studied. An gas average mixture with a content of less than 4% vol in the presence of CeNi1Pd1 was produced, a rather low result and similar to that reported for the gasification of biomass and natural gas.

## Figures and Tables

**Table 1:** Estimated basic properties of synthesized CeO_2_-based nanocatalysts [38].

Sample	S_BET_ ± 0.1 m^2^·g^−1^	dp (nm ± 0.2 nm)				Dispersion (%)	
		NiO	Co_3_O_4_	Fe_2_O_3_	PdO	Ni/Co/Fe	Pd
CeO_2_	67.0	-	-	-	-	-	-
CeNi1Pd1	63.8	6.4	-	-	3.9	12.7	38.6
CeFe1Pd1	64.1	-	-	5.4	6.9	11.2	12.8
CeCo1Pd1	64.4	-	1.9	-	6.1	18.1	20.4

**Table 2:** Atomic content and relationships calculated from the O1s, Ce3d, and Pd3d spectra.

Sample	O (%)	Ce (%)	Pd (%)	Ce^3+^ (%)	O_ads_ (%)	O_latt_ (%)	O_ads_/O_latt_	Pd^2+^ (%)	Pd^0^ (%)
CeO_2_	62.11	37.89	-	18.11	39.44	60.56	0.65	-	-
CeNi1Pd1	58.21	39.79	0.99	32.92	38.21	61.79	0.61	51.22	48.78
CeFe1Pd1	59.29	38.71	0.99	31.11	35.56	64.44	0.55	43.21	56.79
CeCo1Pd1	59.71	38.29	0.99	29.43	31.43	68.57	0.45	38.55	61.45

**Table 3:** Elemental composition percentage of *n*-C_7_ asphaltenes, resins I, and resins II isolated from a Colombian EHO.

Fraction	C (wt%)	H (wt%)	O * (wt%)	N (wt%)	S (wt%)	H/C	$M_{W}\;(g\cdot \mathrm{mol}{-}^{1})$
*n*-C_7_ Asphaltene	81.7	7.8	3.6	<0.5	6.6	1.14	907.3
Resin I	82.1	11.0	<0.5	<0.5	6.1	1.61	609.0
Resin II	80.0	9.1	3.2	1.1	6.6	1.36	957.0

* Obtained by difference.

**Table 4:** Atomic concentrations (%) of (a) oxygen forms, (b) nitrogen forms, (c) carbon forms, and (d) sulfur forms obtained by the fitting results of the O1s, N1s, C1s, and S2p spectra, respectively, for *n*-C_7_ asphaltenes and resins II.

Sample	Peak							
	C1s		O1s		S2p		N1s	
	Assignment	%	Assignment	%	Assignment	%	Assignment	%
*n*-C_7_ Asphaltene	C–C, C–H	77.4	C–O–C, C–O	76.4	Thioether	23.4	Pyridines	40.3
	C=O	18.0	COO–	23.6	Thiophene	74.8	Pyrrolic	32.5
	COO–	4.6			Sulfones	1.8	Amines	27.3
Resin II	C–C, C-H	76.3	C-O–C, C–O	73.2	Thioether	49.5	Pyridines	63.1
	C=O	8.6	COO–	26.8	Thiophene	25.2	Pyrrolic	22.6
	COO–	15.1			Sulfones	25.3	Amines	14.3

**Table 5:** Hydrogen and carbon types present in *n*-C_7_ asphaltenes and resins II fractions obtained by ^1^H NMR and ^13^C NMR analyses, respectively.

Sample	^1^H-NMR				^13^C NMR	
	H_α_	H_β_	H_γ_	H_a_	C_al_	C_ar_
*n*-C_7_ Asphaltene	4.79	62.72	8.98	23.49	35.51	64.48
Resin II	18.09	53.86	17.36	10.69	60.96	39.04

## Supplementary Materials

The following are available online at https://www.mdpi.com/article/10.3390/nano11051301/s1, Figure S1. Softening point calibration curve. Figure S2. Adsorption isotherms constructed for (a) *n*-C_7_ asphaltenes and (b) resins II on CeNi1Pd1 nanoparticles for different R:A ratios. The dotted lines are from the SLE model and the symbols are experimental data. Table S1. Estimated SLE model parameters for adsorption isotherms of *n*-C_7_ asphaltenes and/or resins II on different nanocatalysts and different Resins–Asphaltene (R:A ratios). H represents the Henry’s law constant, K gives an idea about the self-association degree of asphaltenes over nanoparticle surface, and q_m_ is the maximum amount adsorbed. Table S2. Estimated slope and intercept of the linear plots of *q_predicted_* as a function of *q_experimental_* for the adsorption and desorption of *n*-C_7_ asphaltenes in absence and presence of resins II in the systems at different R:A ratios. Section S.1—Solid–Liquid Equilibrium model. Section S.2—Activation energy estimation

## References

[1] Holladay J.D., Hu J., King D.L., Wang Y. (2009). An overview of hydrogen production technologies. Catal. Today.

[2] Navarro R.M., Pena M., Fierro J. (2007). Hydrogen production reactions from carbon feedstocks: Fossil fuels and biomass. Chem. Rev..

[3] Ni M., Leung D.Y., Leung M.K., Sumathy K. (2006). An overview of hydrogen production from biomass. Fuel Process. Technol..

[4] Muradov N. (1993). How to produce hydrogen from fossil fuels without CO_2_ emission. Int. J. Hydrogen Energy.

[5] Seyitoglu S., Dincer I., Kilicarslan A. (2017). Energy and exergy analyses of hydrogen production by coal gasification. Int. J. Hydrogen Energy.

[6] Blok K., Williams R., Katofsky R., Hendriks C.A. (1997). Hydrogen production from natural gas, sequestration of recovered CO_2_ in depleted gas wells and enhanced natural gas recovery. Energy.

[7] Medina O.E., Olmos C., Lopera S.H., Cortés F.B., Franco C.A. (2019). Nanotechnology Applied to Thermal Enhanced Oil Recovery Processes: A Review. Energies.

[8] Santos R., Loh W., Bannwart A., Trevisan O. (2014). An overview of heavy oil properties and its recovery and transportation methods. Braz. J. Chem. Eng..

[9] Guo K., Li H., Yu Z. (2016). In-situ heavy and extra-heavy oil recovery: A review. Fuel.

[10] Curtis C., Kopper R., Decoster E., Guzmán-Garcia A., Huggins C., Knauer L., Minner M., Kupsch N., Linares L.M., Rough H. (2002). Heavy-oil reservoirs. Oilfield Rev..

[11] Rahimi P.M., Gentzis T., Hsu C.S., Robinson P.R. (2006). The chemistry of bitumen and heavy oil processing. Practical Advances in Petroleum Processing.

[12] Hamedi Shokrlu Y., Babadagli T. (2014). Kinetics of the in-situ upgrading of heavy oil by nickel nanoparticle catalysts and its effect on cyclic-steam-stimulation recovery factor. SPE Reserv. Eval. Eng..

[13] Terry R.E., Meyers R.A. (2001). Enhanced oil recovery. Encycl. Phys. Sci. Technol..

[14] Alvarado V., Manrique E. (2010). Enhanced oil recovery: An update review. Energies.

[15] Thomas S. (2008). Enhanced oil recovery—An overview. Oil Gas Sci. Technol. Rev. L’IFP.

[16] Zhang T., Davidson D., Bryant S.L., Huh C. Nanoparticle-stabilized emulsions for applications in enhanced oil recovery. Proceedings of the SPE Improved Oil Recovery Symposium.

[17] Speight J. (2004). Petroleum Asphaltenes-Part 1: Asphaltenes, resins and the structure of petroleum. Oil Gas Sci. Technol..

[18] Franco C.A., Lozano M.M., Acevedo S., Nassar N.N., Cortés F.B. (2015). Effects of resin I on Asphaltene adsorption onto nanoparticles: A novel method for obtaining asphaltenes/resin isotherms. Energy Fuels.

[19] Ali S. (1974). Current status of steam injection as a heavy oil recovery method. J. Can. Pet. Technol..

[20] Medina O.E., Hurtado Y., Caro-Velez C., Cortés F.B., Riazi M., Lopera S.H., Franco C.A. (2019). Improvement of Steam Injection Processes through Nanotechnology: An Approach through In Situ Upgrading and Foam Injection. Energies.

[21] Shin H., Polikar M. (2007). Review of reservoir parameters to optimize SAGD and Fast-SAGD operating conditions. J. Can. Pet. Technol..

[22] Melcon S. (1965). Oil Recovery by In Situ Combustion. https://patents.google.com/patent/US3360041.

[23] Sun X., Zhang Y., Chen G., Gai Z. (2017). Application of nanoparticles in enhanced oil recovery: A critical review of recent progress. Energies.

[24] Nassar N.N., Hassan A., Pereira-Almao P. (2011). Application of nanotechnology for heavy oil upgrading: Catalytic steam gasification/cracking of asphaltenes. Energy Fuels.

[25] Ogolo N., Olafuyi O., Onyekonwu M. Enhanced oil recovery using nanoparticles. Proceedings of the SPE Saudi Arabia Section Technical Symposium and Exhibition.

[26] Agista M.N., Guo K., Yu Z. (2018). A State-of-the-Art Review of Nanoparticles Application in Petroleum with a Focus on Enhanced Oil Recovery. Appl. Sci..

[27] Hashemi R., Nassar N.N., Almao P.P. (2014). Nanoparticle technology for heavy oil in-situ upgrading and recovery enhancement: Opportunities and challenges. Appl. Energy.

[28] Cheraghian G., Hendraningrat L. (2016). A review on applications of nanotechnology in the enhanced oil recovery part B: Effects of nanoparticles on flooding. Int. Nano Lett..

[29] Ariza F., Andrés C. (2015). Synthesis and Application of Supported Metallic and Multi-Metallic Oxides Nanoparticles for In-Situ Upgrading and Inhibition of Formation Damage. Ph.D. Thesis.

[30] Nassar N.N., Hassan A., Pereira-Almao P. (2011). Metal oxide nanoparticles for asphaltene adsorption and oxidation. Energy Fuels.

[31] Hosseinpour N., Khodadadi A.A., Bahramian A., Mortazavi Y. (2013). Asphaltene adsorption onto acidic/basic metal oxide nanoparticles toward in situ upgrading of reservoir oils by nanotechnology. Langmuir.

[32] Kazemzadeh Y., Eshraghi S.E., Kazemi K., Sourani S., Mehrabi M., Ahmadi Y. (2015). Behavior of asphaltene adsorption onto the metal oxide nanoparticle surface and its effect on heavy oil recovery. Ind. Eng. Chem. Res..

[33] Zheng X., Li Y., Zhang L., Shen L., Xiao Y., Zhang Y., Au C., Jiang L. (2019). Insight into the effect of morphology on catalytic performance of porous CeO_2_ nanocrystals for H_2_S selective oxidation. Appl. Catal. B Environ..

[34] Maciel C.G., de Freitas Silva T., Hirooka M.I., Belgacem M.N., Assaf J.M. (2012). Effect of nature of ceria support in CuO/CeO_2_ catalyst for PROX-CO reaction. Fuel.

[35] Eaimsumang S., Wongkasemjit S., Pongstabodee S., Smith S.M., Ratanawilai S., Chollacoop N., Luengnaruemitchai A. (2019). Effect of synthesis time on morphology of CeO_2_ nanoparticles and Au/CeO_2_ and their activity in oxidative steam reforming of methanol. J. Rare Earths.

[36] Razeghi A., Khodadadi A., Ziaei-Azad H., Mortazavi Y. (2010). Activity enhancement of Cu-doped ceria by reductive regeneration of CuO–CeO_2_ catalyst for preferential oxidation of CO in H_2_-rich streams. Chem. Eng. J..

[37] Franco C.A., Montoya T., Nassar N.N., Cortés F.B. (2014). Nioand pdo supported on fumed silica nanoparticles for adsorption and catalytic steam gasification of colombian c7asphaltenes. Handbook on Oil Production Research.

[38] Medina O.E., Gallego J., Arias-Madrid D., Cortés F.B., Franco C.A. (2019). Optimization of the Load of Transition Metal Oxides (Fe_2_O_3_, Co_3_O_4_, NiO and/or PdO) onto CeO_2_ Nanoparticles in Catalytic Steam Decomposition of n-C7 Asphaltenes at Low Temperatures. Nanomaterials.

[39] Cardona Rojas L. (2018). Efecto de Nanopartículas en Procesos con Inyección de Vapor a Diferentes Calidades. Master‘s Thesis.

[40] Cardona L., Arias-Madrid D., Cortés F., Lopera S., Franco C. (2018). Heavy oil upgrading and enhanced recovery in a steam injection process assisted by NiO-and PdO-Functionalized SiO_2_ nanoparticulated catalysts. Catalysts.

[41] Delannoy L., El Hassan N., Musi A., Le To N.N., Krafft J.-M., Louis C. (2006). Preparation of supported gold nanoparticles by a modified incipient wetness impregnation method. J. Phys. Chem. B.

[42] Medina O.E., Gallego J., Restrepo L.G., Cortés F.B., Franco C.A. (2019). Influence of the Ce^4+^/Ce^3^+ Redox-couple on the cyclic regeneration for adsorptive and catalytic performance of NiO-PdO/CeO_2_±δ nanoparticles for n-C7 asphaltene steam gasification. Nanomaterials.

[43] Sellers-Antón B., Bailón-García E., Cardenas-Arenas A., Davó-Quiñonero A., Lozano-Castelló D., Bueno-López A. (2020). Enhancement of the Generation and Transfer of Active Oxygen in Ni/CeO_2_ Catalysts for Soot Combustion by Controlling the Ni–Ceria Contact and the Three-Dimensional Structure. Environ. Sci. Technol..

[44] Wrobel G., Sohier M., D’Huysser A., Bonnelle J., Marcq J. (1993). Hydrogenation catalysts based on nickel and rare earth oxides: Part II: XRD, electron microscopy and XPS studies of the cerium-nickel-oxygen-hydrogen system. Appl. Catal. A Gen..

[45] Wang X., Chen J., Zeng J., Wang Q., Li Z., Qin R., Wu C., Xie Z., Zheng L. (2017). The synergy between atomically dispersed Pd and cerium oxide for enhanced catalytic properties. Nanoscale.

[46] Ancheyta J., Centeno G., Trejo F., Marroquin G., Garcia J., Tenorio E., Torres A. (2002). Extraction and characterization of asphaltenes from different crude oils and solvents. Energy Fuels.

[47] López D., Giraldo L.J., Salazar J.P., Zapata D.M., Ortega D.C., Franco C.A., Cortés F.B. (2017). Metal Oxide Nanoparticles Supported on Macro-Mesoporous Aluminosilicates for Catalytic Steam Gasification of Heavy Oil Fractions for On-Site Upgrading. Catalysts.

[48] Nadkarni R., Nadkarni R. (2007). Guide to ASTM Test Methods for the Analysis of Petroleum Products and Lubricants.

[49] Medina O.E., Gallego J., Nassar N.N., Acevedo S.A., Cortés F.B., Franco C.A. (2020). Thermo-Oxidative Decomposition Behaviors of Different Sources of n-C7 Asphaltenes at High-Pressure Conditions. Energy Fuels.

[50] (2006). Standard Test Method for Softening Point of Bitumen (Ring-and-Ball Apparatus). https://www.astm.org/Standards/D36.htm.

[51] Talu O., Meunier F. (1996). Adsorption of associating molecules in micropores and application to water on carbon. AIChE J..

[52] Franco C.A., Zabala R.D., Zapata J., Mora E., Botero O., Candela C., Castillo A. (2013). Inhibited gas stimulation to mitigate condensate banking and maximize recovery in cupiagua field. SPE Prod. Oper..

[53] Nassar N.N., Hassan A., Luna G., Pereira-Almao P. (2013). Kinetics of the catalytic thermo-oxidation of asphaltenes at isothermal conditions on different metal oxide nanoparticle surfaces. Catal. Today.

[54] Medina Erao O.E., Gallego J., Olmos C.M., Chen X., Cortés F.B., Franco C.A. (2020). Effect of Multifunctional Nanocatalysts on n-C7 Asphaltene Adsorption and Subsequent Oxidation under High Pressure Conditions. Energy Fuels.

[55] Moghtaderi B. (2007). Effects of controlling parameters on production of hydrogen by catalytic steam gasification of biomass at low temperatures. Fuel.

[56] Lozano M.M., Franco C.A., Acevedo S.A., Nassar N.N., Cortés F.B. (2016). Effects of resin I on the catalytic oxidation of n-C7 asphaltenes in the presence of silica-based nanoparticles. RSC Adv..

[57] Rakhmatullin I., Efimov S., Tyurin V., Al-Muntaser A., Klimovitskii A., Varfolomeev M., Klochkov V. (2018). Application of high resolution NMR (1H and 13C) and FTIR spectroscopy for characterization of light and heavy crude oils. J. Pet. Sci. Eng..

[58] Schneider P. (1995). Adsorption isotherms of microporous-mesoporous solids revisited. Appl. Catal. A Gen..

[59] Bates M.K., Jia Q., Doan H., Liang W., Mukerjee S. (2015). Charge-transfer effects in Ni–Fe and Ni–Fe–Co mixed-metal oxides for the alkaline oxygen evolution reaction. ACS Catal..

[60] Dong Y.-B., Jin G.-X., Smith M.D., Huang R.-Q., Tang B., zur Loye H.-C. (2002). [Ag_2_(C_33_H_26_N_2_O_2_)(H_2_O)2(SO_3_CF_3_) _2_]⊙ 0.5C_6_H_6_: A Luminescent Supramolecular Silver (I) Complex Based on Metal-Carbon and Metal-Heteroatom Interactions. Inorg. Chem..

[61] Ignasiak T., Kemp-Jones A., Strausz O. (1977). The molecular structure of Athabasca asphaltene. Cleavage of the carbon-sulfur bonds by radical ion electron transfer reactions. J. Org. Chem..

[62] Arias-Madrid D., Medina O.E., Gallego J., Acevedo S., Correa-Espinal A.A., Cortés F.B., Franco C.A. (2020). NiO, Fe_2_O_3_, and MoO_3_ Supported over SiO_2_ Nanocatalysts for Asphaltene Adsorption and Catalytic Decomposition: Optimization through a Simplex–Centroid Mixture Design of Experiments. Catalysts.

[63] Leon O., Rogel E., Espidel J., Torres G. (2000). Asphaltenes: Structural characterization, self-association, and stability behavior. Energy Fuels.

[64] Alvarez-Ramirez F., Ramirez-Jaramillo E., Ruiz-Morales Y. (2006). Calculation of the interaction potential curve between asphaltene-asphaltene, asphaltene-resin, and resin-resin systems using density functional theory. Energy Fuels.

[65] Merino-Garcia D., Andersen S.I. (2004). Thermodynamic characterization of asphaltene− resin interaction by microcalorimetry. Langmuir.

[66] Medina O.E., Caro-Vélez C., Gallego J., Cortés F.B., Lopera S.H., Franco C.A. (2019). Upgrading of Extra-Heavy Crude Oils by Dispersed Injection of NiO–PdO/CeO2±δ Nanocatalyst-Based Nanofluids in the Steam. Nanomaterials.

[67] Alamolhoda S., Vitale G., Hassan A., Nassar N.N., Almao P.P. (2019). Synergetic effects of cerium and nickel in Ce-Ni-MFI catalysts on low-temperature water-gas shift reaction. Fuel.

[68] Luo M.-F., Hou Z.-Y., Yuan X.-X., Zheng X.-M. (1998). Characterization study of CeO_2_ supported Pd catalyst for low-temperature carbon monoxide oxidation. Catal. Lett..

[69] Gradisher L., Dutcher B., Fan M. (2015). Catalytic hydrogen production from fossil fuels via the water gas shift reaction. Appl. Energy.

[70] Coughlin R.W., Farooque M. (1979). Hydrogen production from coal, water and electrons. Nature.

[71] Li S., Cheng Y. (1995). Catalytic gasification of gas-coal char in CO_2_. Fuel.

[72] Hauserman W.B. (1994). High-yield hydrogen production by catalytic gasification of coal or biomass. Int. J. Hydrogen Energy.

[73] Alamolhoda S., Vitale G., Hassan A., Nassar N.N., Pereira Almao P. (2019). Development and characterization of novel combinations of Ce-Ni-MFI solids for water gas shift reaction. Can. J. Chem. Eng..

[74] Vignatti C.I., Avila M.S., Apesteguia C.R., Garetto T.F. (2011). Study of the water-gas shift reaction over Pt supported on CeO_2_–ZrO_2_ mixed oxides. Catal. Today.

[75] De Oliveira Rocha K., Marques C.M.P., Bueno J.M.C. (2019). Effect of Au doping of Ni/Al_2_O_3_ catalysts used in steam reforming of methane: Mechanism, apparent activation energy, and compensation effect. Chem. Eng. Sci..

[76] Shido T., Iwasawa Y. (1993). Reactant-promoted reaction mechanism for water-gas shift reaction on Rh-doped CeO_2_. J. Catal..

