# Supplementary material for: Catalytic Conversion of n-C7 Asphaltenes and Resins II into Hydrogen Using CeO2-Based Nanocatalysts
Source: Nanomaterials (Basel). 2021 May 14;11(5):1301. doi: 10.3390/nano11051301 (PMC8156694; doi:10.3390/nano11051301)
Supplement: Supplementary file 1 [file nanomaterials-11-01301-s001.zip › nanomaterials-1182942-supplementary.pdf]

nanomaterials

Supplementary Materials

Catalytic Conversion of *n*-C7 asphaltenes and resins II into Hydrogen Using CeO2-based Nanocatalysts

Oscar E. Medina [1], Jaime Gallego [2], Sócrates Acevedo [3], Masoud Riazi [4], Raúl Ocampo-Pérez [5], Farid B. Cortés [1,*], and Camilo A. Franco [1,*]

[1] Grupo de Investigación en Fenómenos de Superficie—Michael Polanyi, Departamento de Procesos y Energía, Facultad de Minas, Universidad Nacional de Colombia, Sede Medellín, 050034 Medellín, Colombia; oemedinae@unal.edu.co (O.E.M.)
[2] Química de Recursos Energéticos y Medio Ambiente, Instituto de Química, Universidad de Antioquia UdeA, Calle 70 No. 52-21, 050010 Medellín, Colombia; andres.gallego@udea.edu.co (J.G.)
[3] Facultad de Ciencias, Escuela de Química, Universidad Central de Venezuela, Caracas 1040, Venezuela; socrates.acevedo@gmail.co (S.A.)
[4] Enhanced Oil Recovery Research Center, IOR-EOR Research Institute, Shiraz University, Shiraz 7193616511, Iran; mriazi@shirazu.ac.ir (M.R.)
[5] Centro de Investigación y Estudios de Posgrado, Facultad de Ciencias Químicas, Universidad Autónoma de San Luis Potosí, Av. Dr. Manuel Nava 6, Zona Universitaria, 78210, San Luis Potosí, México; raul.ocampo@uaslp.mx (R.O.P)
* Correspondence: caafrancoar@unal.edu.co (C.A.F.); fbcortes@unal.edu.co (F.B.C)

S.1- Solid-Liquid Equilibrium model

Solid-Liquid Equilibrium model bases on adsorption and association theory of molecules in microporous surfaces, described by the following equations.

$$C = \frac{\psi H}{1 + K\psi} e^{\left(\frac{\psi}{q_m A}\right)} \tag{S1}$$

$$\psi = \frac{-1 + \sqrt{1 + 4K\xi}}{2K} \tag{S2}$$

$$\xi = \frac{q_m q}{(q_m - q)} A \tag{S3}$$

where C (mmol·mol^{-1}) represents the asphaltenes and/or resins concentration, A (m^2·g^{-1}) is the surface area of nanoparticles, H (mmol·mol^{-1}) refers to the affinity between nanoparticles and resins and/or asphaltenes. Similarly, K (mmol·mol^{-1}) indicates the self-association degree of *n*-C7 asphaltenes and/or resins II molecules on the surface of the nanoparticles, and q_m (mol·mol^{-1}) is the maximum adsorption capacity [1].

S.2- Activation energy estimation

Instantaneous reactivity is represented by Equation S4.

$$\frac{d\alpha}{dt} = K_a \exp\left(-\frac{E_a}{RT}\right) f(\alpha) \tag{S4}$$

where, K_a is the Arrhenius pre-exponential factor, R is the ideal gas constant and E_a the effective activation energy. In addition, $d\alpha / dt$ refers to the change in conversion (α) of asphaltenes and/or resins II concerning time. Integration of Equation (S4) bearing in mind isothermal conditions, lead Equation S5:

$$g(\alpha) = K_a \exp\left(-\frac{E_a}{RT}\right) t \tag{S7}$$

Applying natural logarithm on both sides of Equation S5, the expression can be written in linear terms as Equation S6

$$ln\left(t_{a,i}\right) = ln\left(\frac{g(\alpha)}{K_a}\right) + \frac{E_a}{RTi} \tag{S6}$$

By graphing $\ln(t_{a,i})$ against $1/T_i$, effective activation energy values can be obtained by the slope.

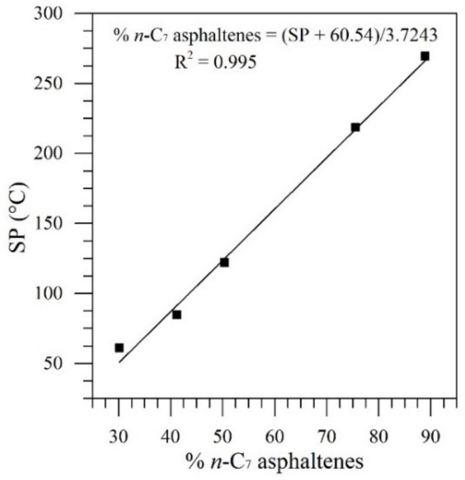

Figure S1. Softening point calibration curve.

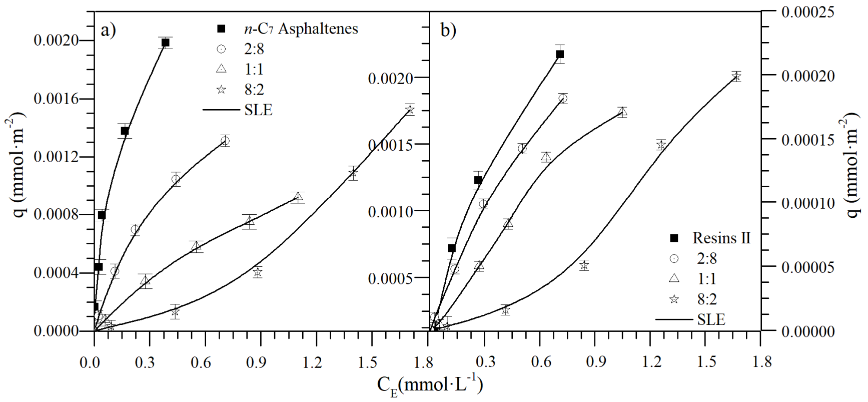

Figure S2. Adsorption isotherms constructed for (a) n-C_7 asphaltenes and (b) resins II on CeNi1Pd1 nanoparticles for different R:A ratios. The dotted lines are from the SLE model and the symbols are experimental data.

Table S1. Estimated SLE model parameters for adsorption isotherms of n-C_7 asphaltenes and/or resins II on different nanocatalysts and different Resins-Asphaltene (R:A ratios). H represents the Henry's law constant, K gives an idea about the self-association degree of asphaltenes over nanoparticle surface, and q_m is the maximum amount adsorbed.

Sample	R:A	n-C_7 asphaltenes H (mmol·g^{-1}) x10^{-3}	K (mmol·g^{-1}) x10^{-3}	q_m (mmol·m^{-2}) x10^{-3}	% RSM	Resins II H (mmol·g^{-1}) x10^{-2}	K (mmol·g^{-1}) x10^{-2}	q_m (mmol·m^{-2})	% RSM
CeNi1Pd1	Individual	6.97	4.36	0.21	0.01	7.60	0.61	0.19	0.01
CeFe1Pd1	Individual	7.25	4.39	0.19	0.01	8.05	0.21	0.18	0.05
CeCo1Pd1	Individual	6.99	6.36	0.21	0.01	9.17	1.66	22.51	0.02
CeO_2	Individual	11.15	22.81	0.13	0.01	11.50	7.96	19.40	0.01
	2:8	8.38	5.75	0.20	0.01	171.68	9.06	23.47	0.02
CeNi1Pd1	1:1	13.90	6.24	0.15	0.01	0.18	8.53	31.57	0.01
	8:2	117.05	6.91	28.18	0.01	4.42	3.35	0.25	0.02

Table S2. Estimated slope and intercept of the linear plots of $q_{predicted}$ as a function of $q_{experimental}$ for the adsorption and desorption of n-C_7 asphaltenes in absence and presence of resins II in the systems at different R:A ratios.

R:A Ratio	m	b (x10^{-3})	R^2
n-C_7 Asphaltenes	1.0	0.0	1.0
2:8	1.03	-1.11	0.99
1:1	1.05	-2.80	0.98
8:2	1.04	-3.21	0.99

References

1. Montoya, T.; Coral, D.; Franco, C.A.; Nassar, N.N.; Cortés, F.B. A novel solid–liquid equilibrium model for describing the adsorption of associating asphaltene molecules onto solid surfaces based on the "chemical theory". *Energy & Fuels* **2014**, *28*, 4963-4975.
